# Supplementary material for: Basic research investment, innovation capability improvement, and economic growth efficiency
Source: PLoS One. 2022 Dec 13;17(12):e0278943. doi: 10.1371/journal.pone.0278943 (PMC9746962; doi:10.1371/journal.pone.0278943)
Supplement: Attachment — Submitted filename: Response to Reviewers.docx [file pone.0278943.s003.docx]

**Revised explanation of 《Basic Research Investment, Innovation Ability Improvement and Economic Growth Efficiency》**

**Dear Review Expert/Editorial Teacher**,

Hello! Thank you very much for your constructive suggestions for revision of the paper. Each of your review comments is an important help in revising and improving this article. After receiving the revision opinions sent by your publication, the author immediately re-consulted the relevant literature and materials, and revised and supplemented according to the opinions of the review experts. The relevant changes are described as follows:

**Reviewer #1**

**Q1**: Literature review must be updated:10.1016/j.jup.2021.101256;10.1080/15567249.2020.1868622.

**Modification instructions**: The author attaches great importance to expert advice. According to experts' opinions. Authors carefully read the above two papers, deepened their understanding of ICT penetration, power consumption, environmental pollution, urbanization and economic growth, and added the above two papers in the literature review section of the paper.

**Q2**: Descriptive statistics are incomplete.

**Modification instructions**: Thanks very much for the opinions of the reviewers. The author adjusted the descriptive statistics of the main variables (Table 1) according to the opinions of the reviewers to make the contents of the table complete.

**Q3**:The structure of the manuscript is confusing: Table 2 should be placed before Table 1...

**Modification instructions**: Based on expert opinion, the author rearranged the structure and position of the chart. And all the tables and figures involved in the article are checked, and strive to make the structure of the article clear, not confusing.

**Q4**: Comparisons with previous results, discussion, Conclusions, policy implications, limitations of the results are too short.

**Modification instructions**: Many thanks to the experts for their valuable advice. According to the expert opinion, the author added the discussion of the existing literature in the corresponding part of the paper, and made a comprehensive and detailed revision to the conclusion, policy suggestion and article deficiency, so as to make the content more abundant and the logic more smooth. It is hoped that the modification results can meet the requirements of experts.

**Q5**: Is the manuscript presented in an intelligible fashion and written in standard English?

**Modification instructions**: According to the expert opinion, the author revises and adjusts the language expression of the full text, and strives to convey the author's views accurately and clearly.
